# Relative accuracy of social and medical determinants of suicide in electronic health records

**DOI:** 10.1111/1475-6773.13540

**Published:** 2020-09-02

**Authors:** Farrokh Alemi, Sanja Avramovic, Keith D. Renshaw, Rania Kanchi, Mark Schwartz

**Affiliations:** ^1^ Department of Health Administration and Policy George Mason University Virginia; ^2^ Department of Population Health New York University New York; ^3^ Department of Psychology George Mason University Virginia; ^4^ Veteran Administration New York Harbor Healthcare System New York

**Keywords:** observational data/quasi‐experiments, social determinants of health, mental health, VA health care system

## Abstract

**Objective:**

This paper compares the accuracy of predicting suicide from Social Determinants of Health (SDoH) or history of illness.

**Population Studied:**

5 313 965 Veterans who at least had two primary care visits between 2008 and 2016.

**Study Design:**

The dependent variable was suicide or intentional self‐injury. The independent variables were 10 495 International Classification of Disease (ICD) Version 9 codes, age, and gender. The ICD codes included 40 V‐codes used for measuring SDoH, such as family disruption, family history of substance abuse, lack of education, legal impediments, social isolation, unemployment, and homelessness. The sample was randomly divided into training (90 percent) and validation (10 percent) sets. Area under the receiver operating characteristic (AROC) was used to measure accuracy of predictions in the validation set.

**Principal Findings:**

Separate analyses were done for inpatient and outpatient codes; the results were similar. In the hospitalized group, the mean age was 67.2 years, and 92.1 percent were male. The mean number of medical diagnostic codes during the study period was 37; and 12.9 percent had at least one SDoH V‐code. At least one episode of suicide or intentional self‐injury occurred in 1.89 percent of cases. SDoH V‐codes, on average, elevated the risk of suicide or intentional self‐injury by 24‐fold (ranging from 4‐ to 86‐fold). An index of 40 SDoH codes predicted suicide or intentional self‐injury with an AROC of 0.64. An index of 10 445 medical diagnoses, without SDoH V‐codes, had AROC of 0.77. The combined SDoH and medical diagnoses codes also had AROC of 0.77.

**Conclusion:**

In predicting suicide or intentional self‐harm, SDoH V‐codes add negligible information beyond what is already available in medical diagnosis codes.

**Implications for Practice:**

Policies that affect SDoH (eg, housing policies, resilience training) may not have an impact on suicide rates, if they do not change the underlying medical causes of SDoH.

**What was known prior to this article?**

- Social determinants (eg, homelessness, unemployment) and mental illness (eg, depression, history of injury) were associated with suicide. Inside electronic health records, V‐codes indicate social determinants of suicide.

**What does this paper contribute?**

- An index based on 40 social determinants of health (measured using V‐codes in electronic health records) accurately predicted probability of suicide, but it did not add any new information beyond what can be inferred from medical diagnoses.

## 1 INTRODUCTION

After decades of research, our ability to predict suicides through clinical interviews or questionnaires remains weak. Two recent comprehensive meta‐analyses of hundreds of studies ^1^, ^2^ found that even some of the most often cited clinically assessed risk factors, such as suicidal ideation, prior attempts, and hopelessness, offer only weak prediction of future attempts or suicide completions. Frustration with lack of predictability of suicides has led some to call for abandoning suicide risk assessment entirely.^3^ The US Preventive Services Task force does not recommend screening for suicide on grounds that screening leads to many false positives; and for those at elevated risk, it is not clear that any treatment reduces the rate of suicides.^4^

The Department of Veterans Affairs (VA) represent the frontier of the field in preventing suicides. Since the 1970s, it has invested heavily in suicide prevention programs. These have included universal risk assessment, comprehensive suicide assessments for patients at risk, clinical review of care for high‐risk patients, in‐home gun lock‐up program, mental health services through team‐based primary care, and suicide hotlines. The effort to prevent suicide is comprehensive, includes every veteran, and is nationwide in scope. It is puzzling and disheartening that the net impact of these extensive and widespread efforts has been no decline in suicides among veterans.^5^

One possible solution that might be worth exploring is to engage in strategies that address social determinants of suicide. The approaches that rely on reducing suicide through changing social determinants are often referred to as resilience training^6^, ^7^ and are part of connecting the veterans to their community. The idea is that if the veteran has a strong community, then the community can help materially and emotionally to reduce the consequences of adverse events. In addition, one may address social determinants by policies that prevent or reduce life stressors, such as food insecurity or homelessness. The literature suggests a strong relationship between social determinants of health (SDoH) and suicides.^8^ The idea of community‐wide suicide prevention that goes beyond medical needs has been explored in the United States Air Force with reported success,^9^, ^10^ albeit not recently.^11^ The purpose of this paper is to establish the relative importance of social and medical determinants of suicide and thus provide insights into when resilience training might help.

It is relatively easy to find medical predictors (eg, depression) of suicide or intentional self‐injury in electronic health records (EHR). In contrast, EHRs do not sufficiently address SDoH.^12^ Within the International Classification of Diseases (ICD version 9), V‐codes include measures of SDoH. In 2017, Torres and colleagues^13^ organized selected V‐codes into 9 categories: (1) Family alcohol/drug addiction, (2) victim or perpetrator of physical or emotional abuse, (3) Family and support group problems, (4) caregiver related problems, (5) education and literacy issues, (6) unemployment or occupational maladjustment, (7) housing issues, (8) legal issue, and (9) other psychosocial issues. Such social codes can add valuable information about the context for medical illness.^14^, ^15^

However, V‐codes are rarely used in EHRs, in less than 2 percent of hospitalizations in a national review of 2013 data.^16^ There are many reasons for infrequent use of these codes. These codes are not reimbursed, and therefore, there are no financial incentives to include them in claims. During the clinical encounter, clinicians may not focus on SDoH. If clinicians do not ask, then these issues are not documented within the EHR. Even if clinicians ask, medical record coders may ignore clinician's documentation as they are required to only include conditions that drive reimbursement. A V‐code may only be entered if the SDoH interfered with delivery of the clinical care, which may be hard to ascertain. At the same time, these same limitations make the codes highly specific to delivery of care and, perhaps, predictive of outcomes such as suicide. To date, the relationship between the V‐codes and suicides has not been reported. This paper addresses that gap.

## 2 METHODS

We selected all US veterans who in the years 2008 through 2016 had at least two primary care visits. We examined all data (including years prior to 2008) for these veterans available through Veterans Affairs Informatics and Computing Infrastructure.^17^ Within these data, we analyzed hospitalization and outpatient codes separately. When a patient is hospitalized, up to 15 codes are listed as reasons for the visit, including anything that affected care during hospitalization. Outpatient visits tend to have fewer codes, typically one code per visit. Thus, hospitalization allows for more opportunities to include V‐codes. During the study time period, 1 986 306 veterans had at least one hospitalization and 5 904 406 had at least one outpatient visit.

The dependent variable was the presence of any one of 48 suicide or intentional self‐injury codes, organized by the Agency for Healthcare Quality and Research.^18^ These codes do not represent all suicides or intentional self‐injuries within these years, as many suicides are not reported.^19^ Thus, these codes likely reflect an underestimate of the true rate of suicide or intentional self‐injury events.

The independent variables in this study were SDoH, age, gender, and illness history. SDoH were measured by 40 unique binary V‐codes, first reported by Torres and colleagues.^11^ A list of these V‐codes is provided in Table 1. Age and gender were recorded at the date of the first reported V‐code.

**TABLE 1:** Likelihood ratio of suicide for hospitalized veterans

Index of stressful life events	EHR‐based social determinants (Version 9 Code, Maps to Version 10 Code)	#of Cases	Suicide or self‐injury	Likelihood ratio
Homelessness & Housing Events	Lack of housing (V60.0, Z59.0)	115 017	29 950	18.65
	Specified housing/economic circumstances (V60.89, Z59.8)	3630	1190	25.83
	Inadequate housing (V60.1, Z59.1)	3362	1120	26.46
	Person living in residential institution (V60.6, Z59.3)	1769	272	9.62
	Unspecified housing or economic circumstance (V60.9, Z59.9)	4096	1522	31.32
Isolation	Person living alone (V60.3, Z60.2)	7758	1182	9.52
	No other household member able to render care (V60.4, Z74.2)	5026	386	4.41
	Social maladjustment (V62.4, Z60.3)	13 147	4880	31.27
Arguments with Spouse	Counseling for marital & partner problems (V61.10, Z71.89)	10 890	4412	36.07
	Interpersonal problems not elsewhere indicated (V62.81, Z65.8)	10 754	4238	34.45
	Counseling for victim of spousal and partner abuse (V61.11, Z69.11)	91	48	59.12
Unemployed	Unemployment (V62.0, Z56.0)	85 085	26 510	23.97
Changes in Business/Work	Other occupational circumstances/maladjustments (V62.29, Z56.1)	3919	1476	32.00
	Adverse effects of work environment (V62.1,Z56.9)	532	196	30.90
Poverty	Inadequate material resources (V60.2, Z59.5)	32 770	12 474	32.55
Court Involved	Legal circumstances (V62.5, Z65.3)	19 214	6728	28.54
Family Disruption	Family disruption due to divorce/legal separation (V61.03, Z63.5)	3345	1310	34.10
	Family disruption due to military deployment (V61.01, Z63.32)	125	62	52.13
	Problems with aged parents or in‐laws (V61.3, Z63.79)	114	34	22.51
	Family disruption due to parent‐child estrangement (V61.04, Z63.8)	268	140	57.93
	Family disruption due to child in foster care (V61.06, Z63.32)	50	24	48.89
	Family disruption due to child welfare custody (V61.05, Z63.32)	39	8	13.67
	Family disruption due to military deployment return (V61.02, Z63.8)	46	14	23.17
	Counseling for parent‐biological child problem (V61.23, Z71.89)	58	22	32.37
	Counseling for parent‐child problem, unspecified (V61.20, Z71.89)	553	230	37.72
	Other parent‐child problems (V61.29, Z62.1)	340	150	41.82
School Events	Educational circumstances (V62.3, Z55.9)	572	228	35.11
Not Listed in Stressful Life Events But Listed in V‐codes	Holiday relief care (V60.5, Z75.5)	9959	344	1.89
	History of physical abuse (V15.41, Z91.410)	9068	3814	38.45
	Other psychological/physical stress (V62.89, Z65.8)	6466	2138	26.16
	Return from military deployment (V62.22, Z65.5)	729	208	21.15
	History of emotional abuse (V15.42, Z91.411)	1179	516	41.22
	Unspecified psychosocial circumstance (V62.9, Z65.9)	2679	924	27.89
	History of other psychological trauma (V15.49, Z91.49)	458	174	32.45
	Alcoholism in Family (V61.41, Z63.72)	404	138	27.48
	Substance abuse in family (V61.42, Z63.79)	112	46	36.92
	Counseling for victim of child abuse (V61.21, Z69.010)	152	68	42.88
	Counseling for perpetrator of physical/sexual abuse (V62.83, Z69.021)	55	34	85.75
	Foster care (status) (V60.81, Z62.21)	43	8	12.11
	Current military deployment status (V62.21, Z65.8)	17	0	18.00

Notes

Stressful Life Events index includes these additional factors not in V‐codes: new disability, addition or loss of a child, death of a friend, foreclosure, and change in church. In addition, sexual dysfunction, pregnancy, and sleep disruptions are also not listed in V‐codes but are available in the medical record. Version 10 codes are approximate mapping from version 9 codes.

Medical history was captured through 10 445 ICD‐9 diagnostic codes for medical and mental health disorders. Each of these codes was used as binary predictor. If the disease was present, the code was assigned a value of 1, and if it was not reported, it was assigned a value of 0. The presence of such a large number of independent variables is reasonable, because the size of data is several folds larger than the number of independent variables.

For the analysis, the study sample was randomly divided into training (90 percent of data) and validation sets (10 percent of data). Likelihood ratios (LR) were estimated in the training data, and the accuracy of the predictions was tested in the validation set. For each diagnostic code, we calculated the likelihood ratio of suicide or intentional self‐injury in the training set as follows:

$${\text{LR}}_{\text{Code}}=\left\{\frac{\begin{array}{c} \frac{1}{\text{Sample size}\;+\;\text{1}}\;\text{When none with code commit suicide}\\ \text{Sample size}\;+\;\text{1}\;\text{When all with code commit suicide}\\ \text{Prevalence of code in patients who committed suicide} \end{array}}{\text{Prevalence of code in patients who did not commit suicide}}\right.$$

Likelihood ratios indicate how many times the odds of suicide or intentional self‐injury changes, if the patient has the code. A likelihood ratio above one increases the odds of suicide and a ratio below one decreases it.

Many statisticians add 0.5 to number of cases to avoid division by zero or having a likelihood ratio of zero. This approach does not provide an adjustment that is proportional to the sample of patients with the diagnosis. Obviously, as sample size increases we have more confidence in large likelihood ratios. To have an adjustment that is proportional, we calculated the likelihood ratio for diagnoses where no patient commits suicide as 1/(sample size + 1). We calculated the likelihood ratio for diagnoses where every patient commits suicide as (sample size + 1). This removes the division by zero, and zero likelihood ratios, in a way that is proportional to sample size. For example, a diagnoses in which no one committed suicide and 17 people did not commit suicide would be assigned a likelihood ratio of 1/(17 + 1).

Naïve Bayes was used to estimates odds of suicide or intentional self‐injury for patients with multiple social or medical illness codes. To calculate the change in odds of suicide or intentional self‐injury for patients in the validation set, we used the likelihood ratios associated with codes in the patient's record:

$$\text{Change in Suicide Odds}\;=\;\prod {\text{LR}}_{i}$$

Naïve Bayes is similar to linear logistic regression,^20^ as it assumes independence and allows no interaction among diagnostic codes. Despite this false assumption of independence, in large sparse data, such as the data in this study, naïve Bayes has been relatively accurate in predicting patient outcomes.^21^, ^22^, ^23^

The accuracy of the predictions was calculated using area under the receiver operating characteristic (AROC) curves. An AROC of 100 percent would indicates perfect prediction, and an AROC of 50 percent would indicate random prediction. We determined separate AROC curves to predict suicide in the training set for (a) the 40 codes related to SDoH, (b) for the 10 455 codes related to medical illness or injury but without the 40 social determinants, and (c) for all 10 495 combined set of codes. This type of hierarchical analysis of accuracy follows the common regression practice of first starting with one set of variables; then in the next model, including additional predictors of interest. This approach allows one to see if social determinants of suicide affect accuracy above and beyond the effect of injury/illness predictors. The models developed in the training set were then tested in the validation set.

## 3 RESULTS

The results for inpatient and outpatient codes were similar, to save space we focus on inpatient codes. Details of outpatient codes are available through Appendix S1. There were 1 986 306 unique veterans with at least one hospitalization, 90 percent of whom were in the training set. The mean (SD) age of the sample was 67.2 years (standard deviation, SD = 16.4), and 92.1 percent were male. Within the training set, 33 126 (1.89 percent) committed suicide or intentional self‐injury. Suicides occurred in 19‐year‐olds and 90‐year‐olds.

The V‐codes were more frequent than previously reported in the literature; 12.9 percent of the veterans had at least one V‐code. The most frequently used code was “Lack of housing (V60.0),” which was reported for 115 017 veterans, or 6.43 percent of the training sample. The remaining social codes were used infrequently, with a low of only 17 patients with code for “Persons currently in military deployment.” The mean (SD) number of medical diagnostic codes was 37 (SD = 28.9), ranging from 1 to 323.

Despite being relatively rare, social determinants of suicide were predictive of suicide or intentional self‐injury. On average, these codes increased the odds of suicide or intentional self‐injury by 24‐fold (ranging from 4‐fold to 86‐fold). For example, lack of housing increased the odds of suicide or intentional self‐injury by 18.65 times. “Inadequate housing” affected care of 3362 patients, but it increased the odds of suicide or intentional self‐injury by 26.46 times. Similarly, the code for “legal circumstances that interfere with care” was rare (19 214 out of 1 787 675 or in 1.07 percent of cases) but increased the odds of self‐injury by 28.54‐fold. Table 1 provides a complete list of the 40 codes and the associated likelihood ratio for suicide or intentional self‐injury.

The codes in Table 1 also constitute an EHR‐based index for predicting risk of suicide from SDoH. The likelihood ratios reported in Table 1 show how the index could be scored.

In our training sample, there were 123 499 female patients, 4378 of whom committed suicide or intentional self‐injury. Female patients were 1.88 times more likely to commit suicide or intentional self‐injury. In contrast, there were 1 631 050 male patients, 28 748 committed suicide or intentional self‐injury, with a likelihood ratio of 0.93 compared with female patients.

**FIGURE 1:**
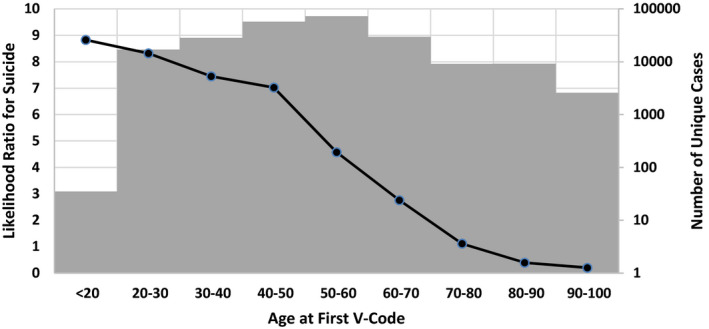
Suicide and age of onset of social determinants [Color figure can be viewed at wileyonlinelibrary.com]

Figure 1 shows the likelihood ratio of suicide or intentional self‐injury at the age of first reported V‐code. These odds are calculated for each decade separately, treating age as many discrete, as opposed to one continuous, variable. If the V‐code was first reported at age less than 20, suicide or intentional self‐injury was 8.82 times more likely than the average veteran in the sample. In contrast, if the V‐code was first reported at age 90, then the likelihood ratio associated with suicide or intentional self‐injury was 0.2, meaning that suicide was five times less likely than the average veteran.

**FIGURE 2:**
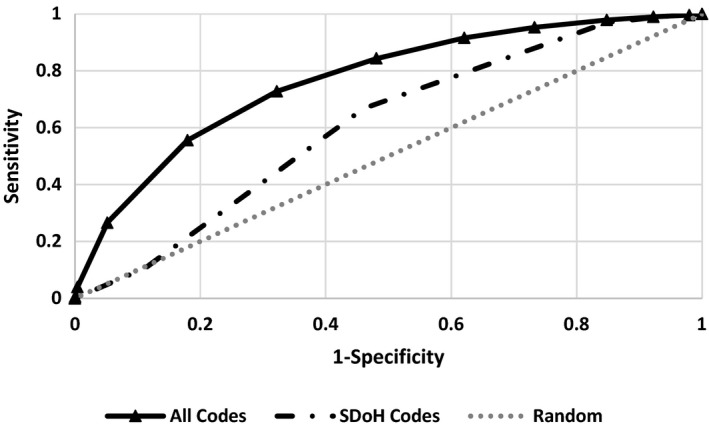
Accuracy of predicting suicide/intentional self‐injury

Figure 2 shows the receiver operating curve for predicting suicide or intentional self‐injury in the validation set. The solid line shows the performance of predicting suicide or intentional self‐injury from age of onset, gender, and 40 V‐codes. The area under the receiver operating curve (AROC) was 0.64. In contrast, predicting suicides from 10 495 diagnostic codes (eg, history of self‐injury, psychiatric illness, etc), including 40 V‐codes, had an AROC of 0.77. In other words, a model based on 40 V‐codes for SDoH was 83.12 percent as accurate as a model based all 10 495 codes. This suggests that the 40 V‐codes, by themselves, provide a parsimonious and relatively accurate model for predicting suicides or intentional self‐injuries.

Either the model based on the 40 SDoH codes (AROC = 0.64) or the model based on 10 455 medical illness codes (AROC of 0.77) were accurate predictors of suicide or self‐injury. The model based on the combined SDoH and medical illness codes had an AROC of 0.77. Adding SDoH codes to medical illness codes had a negligible independent effect on accuracy of predictions (AROC of 0.77 did not change). This suggests that no new insight is gained from 40 social determinants, if a comprehensive set of 10 455 illness/injury predictors are in the model.

## 4 EHR‐BASED SOCIAL DETERMINANTS OF HEALTH INDEX

The 40 coded social determinants, gender, and age (and the associated likelihood ratios) constitute an index of how social determinants may affect the rate of suicide or intentional self‐injury. To score this index, first multiply all likelihood ratios associated with age of the patient, gender of the patient, and each of their V‐codes by each other. Then, multiply by prior odds of suicide, a constant calculated to be 0.0189 in this sample. This formula produces the odds of suicide or intentional self‐injury. To calculate the probability of suicide, divide the odds by one plus the odds.

Table 2 shows these calculations for an example of one patient. This male patient became homeless for the first time at age 55. He had 10 diagnoses that were reported in EHR but ignored by the SDoH index for suicide. These diagnoses, while important in predicting suicide, are not part of the SDoH. The likelihood ratio using first onset, homelessness, and gender was obtained from Table 1 and Figure 1. These were, respectively, 4.58, 18.65, and 0.93. The net impact of these factors was estimated by multiplying the likelihood ratios with each other. The odds of suicide is a product of the likelihood ratios and the prior odds. The prior odds of suicide was 0.0192, and therefore, the odds of suicide after considering the 3 factors was 1.52. The probability of suicide can be calculated from odds/(1 + odds), and in this case, it was 1.52/(1 + 1.52) = 0.60. Therefore, in this example, the probability of suicide increased substantially from 2 percent to 60 percent.

**TABLE 2:** Calculation of SDoH suicide scores for a hospitalized patient

Patient ID 1		Score
1. 10 non‐V‐code diagnoses (ignored as these are not social determinants of health)		1.00
2. Age	First onset of V‐code at 55	4.58
3. Gender	Male	0.93
4 V‐codes	V60.0 Lack of housing	18.65
	No other	1.0
5. Product of likelihood ratios, 1.00*4.58*0.93*18.65*1.0 = 79.40		79.40
6. Prior odds of suicide		0.02
7. Odds of suicide, 79.4*0.02 = 1.52		1.52
8. Predicted probability of suicide, 1.52/(1 + 1.52) = 0.60		0.60

## 5 PREDICTING SUICIDE FROM ILLNESS HISTORY

In Table 1, we presented the likelihood ratios associated with SDoH. In Appendix S1, we provide the likelihood ratios associated with 10 455 illness/injury risk factors. These likelihoods cover a great range of values. Some diagnoses increase (eg, previous overdose of antidepressants), and other diagnoses decrease (eg, anemia in neoplastic disease), the probability of suicide. The likelihood ratios provided online can be used to predict suicides from patients’ medical history. In any particular case, usually a handful of the 10 455 medical diagnoses are present. The prior odds of suicide and the likelihood ratios associated with the codes in the patient's medical history are multiplied together to get the posterior odds of suicide. Appendix S1 can be used by EHR vendors to risk‐rate patients for suicide or intentional self‐harm.

## 6 DISCUSSION

In a national sample of US veterans from 2008 to 2016, 40 SDoH V‐codes were used relatively infrequently (12.9 percent). These codes described a variety of social risk factors, including family disruption, isolation, family addiction, lack of education, legal impediments, unemployment, and lack of housing. Although seldom used, these social codes were very informative. These codes, when present, increased the odds of suicide, on average, by 24 folds. A model predicting from these 40 codes accurately predicted suicide in validation sample (AROC of 0.64). The relatively infrequent SDoH codes predict, even more rare, suicides or intentional self‐injuries.

A model based on medical illness codes, not including the 40 SDoH codes, also accurately predicted suicide or self‐injury (AROC = 0.77). To put this finding in context, it is instructive to compare it with previous efforts to predict suicide from data in electronic health records. Tran and colleagues classified 140 000 patients’ diagnoses into 202 broad categories, which were used to predict suicide attempts. The study found an area under receiver operating curve (AROC) of 79 percent.^24^ In 2015, McCarthy and colleagues used a stratified matched case‐control study design to evaluate impact of risk factors found in VA’s electronic health records. They settled on 36 risk factors.^25^ The model showed promise in identifying patients in the 1 percent highest‐risk category for suicide. The VA Office of Suicide Prevention used LASSO regression to predict suicides accurately with 61 risk factors.^26^ In 2017, Walsh et al reported a machine learning predictive model that accurately predicted suicide attempts (AROC of 84 percent) and improved detection from 720 to 7 days before the suicide attempt, and despite using data in electronic health record had predictions that shifted across time.^27^ In 2018, investigators using data from Kaiser Health Plans developed predictive models for suicide. They reported AROC of 86 percent.^28^ None of the referenced took into account the social V‐codes.

**FIGURE 3:**
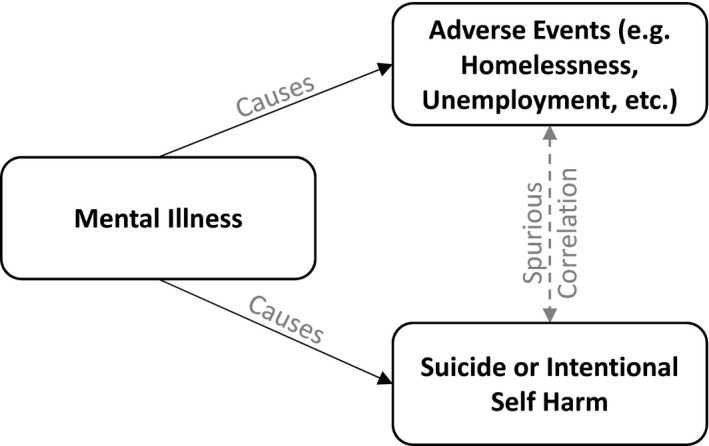
Mental health causes suicide and other adverse social events

When comparing the performance of the two models, medical diagnoses were a more accurate predictor of suicide or intentional self‐injury than SDoH variables. Furthermore, when medical diagnoses were included in the model, SDoH V‐codes did not add additional predictive accuracy. This suggests that impact of SDoH V‐codes and medical diagnoses on accuracy might be confounded. One possible explanation of these data is that mental illness causes both suicide and adverse social events such as family dissolution, homelessness, or unemployment (see Figure 3).^29^ Then, the correlation between SDoH codes and suicide may be spurious and a reflection that both of these events have a common cause. For example, it is generally believed, and the data in this paper supports, that homelessness is associated with suicide. It has a large likelihood ratio, indicative of strong association. Yet, studies that randomly reduced homeless do not report reduction in suicide.^30^ Mental illness causes homelessness. Mental illness also causes suicide. The correlation between homelessness and suicide may be an artifact of mental illness. In randomized studies, where mental illness is controlled for, the correlation between homelessness and suicide disappears; but in observational data, where the common cause is not controlled, the correlation exists.

A by‐product of our work has been the development of an EHR‐based index of SDoH. It may be helpful to compare this to the index of Social Readjustment Rating Scale, which also builds on stressful life events.^31^, ^32^ Table 1 shows the events scored in both scales. While each index defines the variables differently, the following items are present in both: family dissolution (for various reasons), court involvement, unemployment, economic problems, lack of material success, social isolation, homelessness/housing issues, school‐related stress (start/end), business or work‐related events, and problems with spouse. Several variables are in the Social Readjustment Rating Scale and not in the EHR‐based list of social determinants, even though some of these data are available inside EHR and can be added to the EHR list of social determinants. These include death of spouse/close friend, new disability, addition or loss of a child, sexual dysfunction, pregnancy, change in sleep patterns, foreclosure or new loan mortgage, and major change in social/church/recreation activities. There are also variables in the EHR‐based index that are not in the Social Readjustment Rating Scale: history of family addiction, history of child abuse, spousal/partner abuse, perpetrator or victim of abuse, multiple military deployment issues, and social/interpersonal problems. The utility of our EHR‐based index should be tested further in other databases before it is used in clinical settings.

This study is limited in several ways. The study was done on veterans and may not generalize to the US population. This study focusses on reported suicide or reported intentional harm. Many suicides are not reported. Future studies should include unreported suicides, if possible. This study used social determinants of suicide that were available in the EHR and that corresponded closely to life stresses that have received support in the literature. A number of important social determinants such as food security, access to green spaces, and social pressure were not included in our analysis because they are not available as V‐codes. The more comprehensive list of social codes in ICD‐10 (Z‐codes), and their more frequent use in recent years, may be more predictive of suicide. In studies of suicide, one often faces limited data. Therefore, it is difficult to conduct subgroup analysis for women and minorities. This paper has reported the likelihood of suicide, given various social and medical risk factors. The likelihood of suicide measures should not be interpreted as causal impact of the risk factor on suicide. A likelihood ratio measures the association between suicide and the risk factor, and correlation is not causation.

Future studies should examine whether various social codes have a differential impact in women and minority subgroups. The literature suggests that women and minorities have different rates of, and use different means of, suicide. It may be possible that social determinants, like sexual abuse, affect women and minorities differently. Future research in this area could extend the prediction from social codes to other illnesses besides suicide. For instance, it may be useful to study the association between social codes with obesity, diabetes,^33^ and COVID‐19.^34^ Now that the potential value of social codes in predicting suicide is evident, it is important to continue to study these variables, as the use of these codes increases.

## CONFLICT OF INTEREST

None.

## Supporting information

Supplementary Material

Appendix S1
